# The Impact of Public Benefits on Low-Income Older Adults’ Financial Well-Being

**DOI:** 10.1093/geroni/igaa057.2601

**Published:** 2020-12-16

**Authors:** Lauren Popham

**Affiliations:** National Council on Aging, Arlington, Virginia, United States

## Abstract

Nineteen percent (13 million) older adults have incomes below 150% of the Federal Poverty Level, leaving them with limited means to afford basic living expenses. Public benefits can help bridge the gap allowing older adults to afford food, home energy, and health expenses. There are studies demonstrating the positive health outcomes associated with public benefits in older adults. It remains unclear how benefits may also improve subjective measures of well-being in older adults. To examine this question, baseline measures of well-being including the CFPB Financial Well-Being Scale were administered to older adults before they enrolled in benefits and again six months after receiving benefits to examine changes in well-being as a result of accessing benefits to help ease some of their financial burdens. Results revealed that older adults experience subjective, psychological improvements from benefits. These findings have implications for the social and behavioral determinants of health in older adults.

